# Human heart valve-derived scaffold improves cardiac repair in a murine model of myocardial infarction

**DOI:** 10.1038/srep39988

**Published:** 2017-01-04

**Authors:** Long Wan, Yao Chen, Zhenhua Wang, Weijun Wang, Sebastian Schmull, Jun Dong, Song Xue, Hans Imboden, Jun Li

**Affiliations:** 1 Laboratory of Cardiovascular Sciences, Renji-Med X Clinical Stem Cell Research Center, Ren Ji Hospital, School of Medicine, Shanghai Jiao Tong University, China; 2 Department of Cardiovascular Surgery, Ren Ji Hospital, School of Medicine, Shanghai Jiao Tong University, China; 3 German Rheumatism Research Centre, Berlin, Germany; 4 Institute of Cell Biology, University of Bern, Switzerland

## Abstract

Cardiac tissue engineering using biomaterials with or without combination of stem cell therapy offers a new option for repairing infarcted heart. However, the bioactivity of biomaterials remains to be optimized because currently available biomaterials do not mimic the biochemical components as well as the structural properties of native myocardial extracellular matrix. Here we hypothesized that human heart valve-derived scaffold (hHVS), as a clinically relevant novel biomaterial, may provide the proper microenvironment of native myocardial extracellular matrix for cardiac repair. In this study, human heart valve tissue was sliced into 100 μm tissue sheet by frozen-sectioning and then decellularized to form the hHVS. Upon anchoring onto the hHVS, post-infarct murine BM c-kit+ cells exhibited an increased capacity for proliferation and cardiomyogenic differentiation *in vitro*. When used to patch infarcted heart in a murine model of myocardial infarction, either implantation of the hHVS alone or c-kit+ cell-seeded hHVS significantly improved cardiac function and reduced infarct size; while c-kit+ cell-seeded hHVS was even superior to the hHVS alone. Thus, we have successfully developed a hHVS for cardiac repair. Our *in vitro* and *in vivo* observations provide the first clinically relevant evidence for translating the hHVS-based biomaterials into clinical strategies to treat myocardial infarction.

Myocardial infarction (MI), also known as heart attack, leads to the loss of ischemic cardiomyocytes and degradation of myocardial extracellular matrices, characterized by left ventricular (LV) wall thinning and chamber dilation. When ischemically impaired heart is unable to compensate for reduced cardiac output, heart failure results. Accumulating experimental and clinical evidence indicates that intramyocardial transplantation of bone marrow (BM) undifferentiated cells, i.e. c-kit+ stem cells, improves cardiac repair^1^,^2^,^3^,^4^, and that mechanisms including paracrine effects, angiogenesis, transdifferentiation, and cell fusion have been suggested^2^,^3^,^5^,^6^. However, BM stem cell transplantation for repairing infarcted heart is currently hampered by the low rates of cell engraftment and poor cell survival post intramyocardial cell injection^7^,^8^. Studies show that 50–90% of injected cells are lost by extrusion and that 90% of remaining cells die within one week after transplantation because of the lack of nutrition and oxygen supply surrounding the ischemic myocardium^7^,^8^,^9^,^10^. Thus, attempts have been extensively made to improve myocardial cell retention and survival rate, through implanting cells repopulated on a biomaterial scaffold^9^,^11^. So far, cardiac tissue scaffold is designed mainly based on natural and synthetic biomaterials^12^,^13^,^14^,^15^,^16^,^17^,^18^,^19^, which do not mimic the biochemical components and structural properties of native myocardial extracellular matrix. Although implantation of currently available biomaterials, to some extent, increases LV wall thickness and prevents LV dilatation in experimental models of MI the fundamental issue associated with the bioactivity of these biomaterials still remains unsolved^20^. Recent experimental studies reveal that the native extracellular matrix (as a 3D structural scaffold) contains optimal biochemical constituents, facilitating the integration of implanted cells into the host organ/tissue. Here, we reasoned that the human native myocardial tissue-derived matrix might provide a clinically relevant novel scaffold as well as support BM stem cell transplantation for cardiac repair.

In this study, we have successfully developed a human heart valve-derived scaffold (hHVS). We show that the hHVS was able to promote proliferation and cardiomyogenic differentiation of BM c-kit+ cells *in vitro*. Moreover, when used to patch infarcted heart in a murine model of MI, both the hHVS and c-kit+ cell-seeded hHVS preserved cardiac function and reduced myocardial injury, with the latter exerting a more profound effect. Thus, this study presents a novel native myocardial tissue-derived scaffold which is featured with superior cardiac repair function and potential therapeutic benefits.

## Results

### Characterization of hHVS

**Figure 1:**
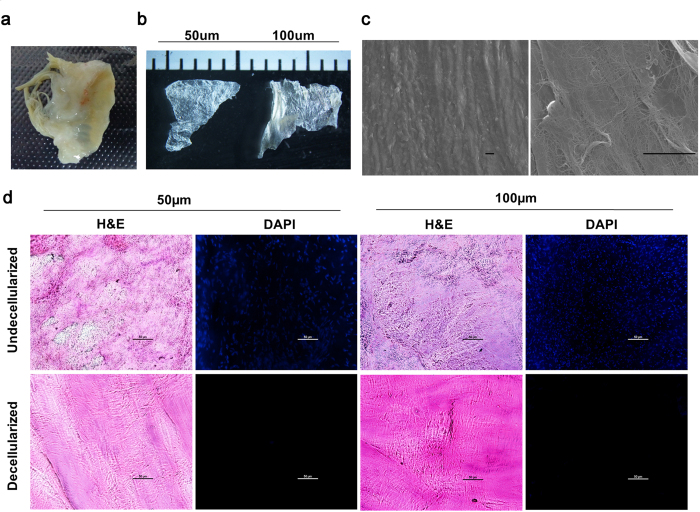
Characterization of hHVS. (**a**) Macroscopic view of human valve tissue before sectioning. (**b**) Macroscopic view of the hHVS sections of 50 μm and 100 μm. (**c**) The 100 μm hHVS under the scanning electron microscope (1000×, 6000×) after decellularization treatment. Scale bar, 20 μm. (**d**) Histological staining of the hHVS (50 μm, 100 μm) before and after decellularization treatment. Hematoxylin and eosin, H&E; DAPI staining for the detection of cellular components/nuclei. Scale bar, 50 μm.

*Ex vivo* human heart valve tissues (Fig. 1a) were sliced into tissue sheets by frozen-sectioning at 50 μm or 100 μm thicknesses (Fig. 1b). Using a modified approach we were able to shorten the duration of decellularization treatment to 12 hours for both 50 μm and 100 μm tissue sheets. In this study, all the experiments were performed using the 100 μm tissue sheet, which was more convenient for *ex vivo* culture. After decellularization, scanning electron microscope images of the hHVS showed interconnected porous meshes with well preserved network architectures of fine nano-fibres and confirmed the removal of cellular components (Fig. 1c). Histological analysis of the hHVS confirmed that the cellular components were completely removed as evidenced by the absence of DAPI stained cellular nuclei for both 50 μm and 100 μm tissue sheets (Fig. 1d).

**Figure 2:**
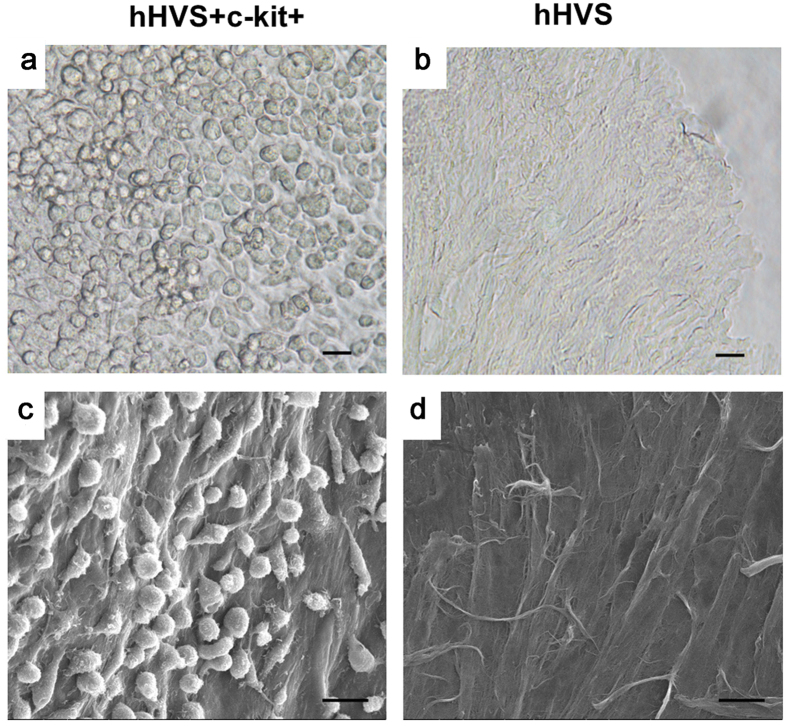
Cell-scaffold adhesion. (**a**) Adhesion of growing BM c-kit+ cells on the hHVS under optical microscope after 10 days in cell culture. (**b**) hHVS without growing cells under the optical microscope. (**c**) Adhesion of growing BM c-kit+ cells on the hHVS under scanning electron microscope after 10 days cell culture. (**d**) hHVS without growing cells under the scanning electron microscope. Scale bar, 20 μm. Data shown are representative of 4 independent experiments.

To evaluate cell-scaffold adhesion, we seeded *ex vivo* murine BM c-kit+ cells (3 × 10^5^ cells/cm^2^) (purity > 85%; Supplementary Fig. S1) onto the hHVS inserted in a 24-well cell culture plate. After 3 days in culture, unattached cells were removed by gentle shaking for more than 30 seconds and adherent cells were counted by flow cytometric analysis. The density of adherent cells was around 9 × 10^4^ cells/well, which was 30% of the initial seeding density of approximately 3 × 10^5^ cells/well. At day 10 of the cell culture, about 2.4 × 10^5^ cells/well were adherent (Fig. 2a,b) and scanning electron microscope images also confirmed the adhesion of growing c-kit+ cells on the surface of the hHVS (Fig. 2c,d), demonstrating a cell retention capacity of the hHVS.

### hHVS Promotes Proliferation of Murine BM c-kit+ Cells

**Figure 3:**
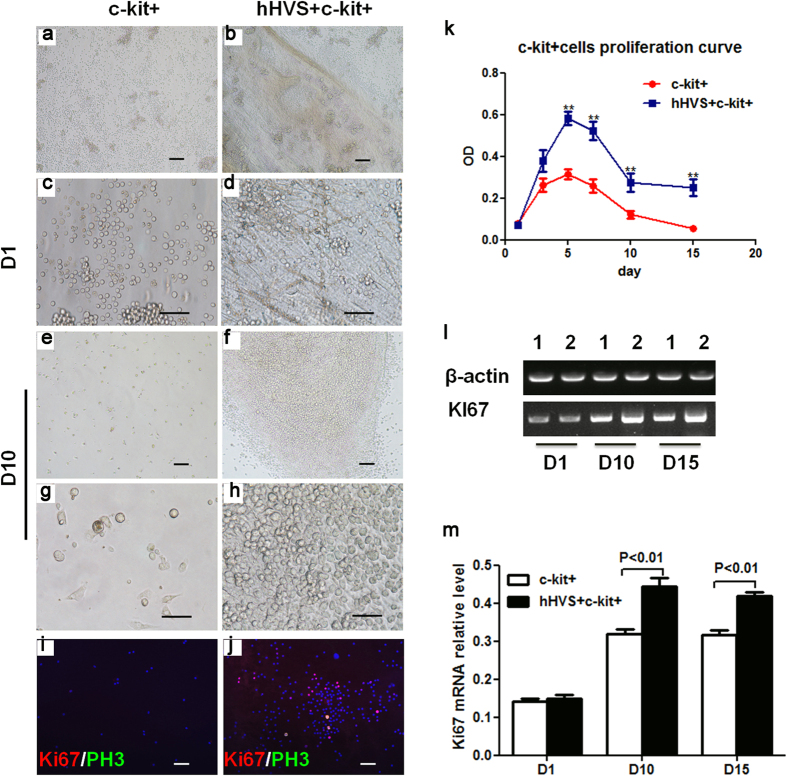
The hHVS promotes proliferation of c-kit+ cells. (**a**–**h**) Proliferation/engraftment of murine BM c-kit+ cells cultured without hHVS (c-kit+) or seeded on the hHVS (hHVS + c-kit+) at indicated time points under optical microscope. Scale bar, 50 μm. Data shown are representative of 3 independent experiments. (**k**) Proliferation of c-kit+ cells seeded on the hHVS or cultured without hHVS (n = 8). (**i**,**j**) Merged immunohistochemical staining of proliferating markers Ki67, PH3 and nuclei (DAPI). Scale bar, 50 μm. (**l**,**m**) RT-PCR (**l**) and semi-qPCR analysis (**m**) for mRNA levels of ki67 in c-kit+ cells seeded on the hHVS or cultured without hHVS on days 1, 10 and 15 (n = 3).

To study the effects of hHVS on cell proliferation, we compared the proliferative behavior of murine BM c-kit+ cells growing on the hHVS to that of the cells cultured without hHVS. An increase in proliferation of the cells attached to hHVS was observed from day 1 to day 5, reaching the maximal level (confluent) on day 5. Subsequently, cell proliferation decreased, ceased, and remained unchanged (from day 10 onward). At all the measured time points (i.e. days 3, 5, 7, 10, and 15), the proliferation of cells attached to the hHVS was much higher than cells cultured without hHVS (Fig. 3a–h,k). Cell proliferation was also shown by increased expression of proliferation markers Ki67/PH3 in cells attached to the hHVS when compared with cells cultured without hHVS (Fig. 3i,j). Reverse-transcription PCR and semi-qPCR analyses further confirmed that the expression of Ki67 in cells attached to the hHVS was upregulated 1.42-fold and 1.35-fold on days 10 and 15, respectively, when compared with cells cultured without hHVS (Fig. 3l,m). Thus, in addition to its ability to retain cells, hHVS also promotes proliferation of cells that attach to it.

### hHVS Induces Cardiomyogenic Differentiation of Murine BM c-kit+ Cells

**Figure 4:**
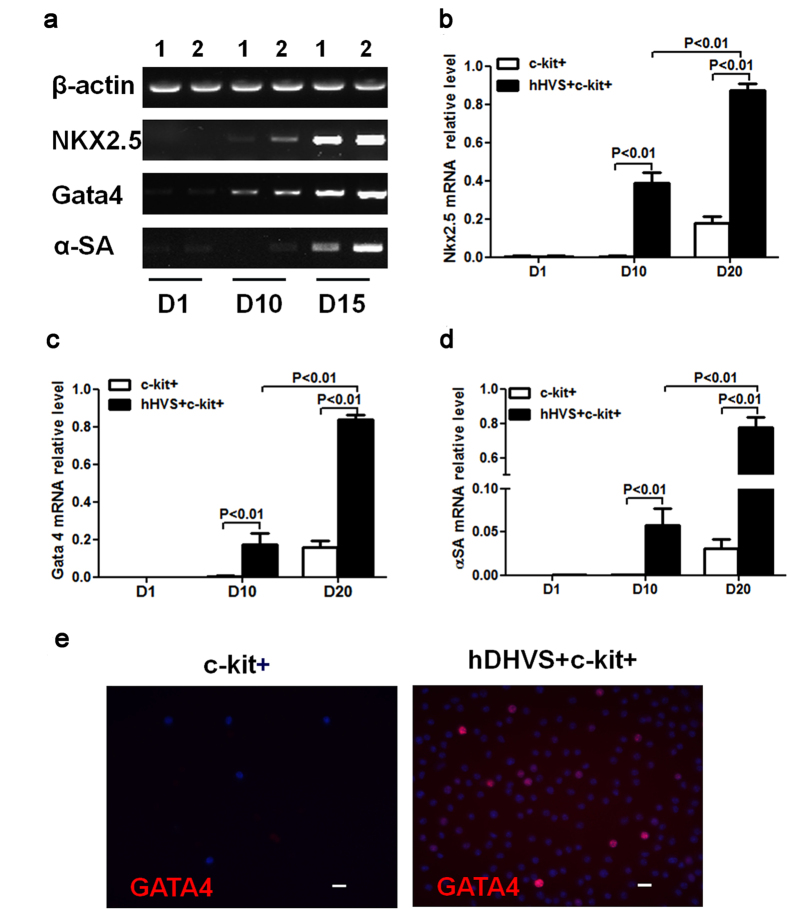
The hHVS promotes cardiomyogenic differentiation of c-kit+ cells. (**a**) RT-PCR showing the expression of cardiomyogenic differentiation markers Nkx2.5, Gata4, and α-SA in c-kit+ cell-seeded on hHVS or cultured without hHVS at indicated time points of cell cultures. (**b**–**d**) Semi-qPCR analysis for relative mRNA levels of Nkx2.5 (**b**), Gata4 (**c**), and α-SA (**d**). Data shown are representative of 3 independent experiments. (**e**) Immunofluorescence staining of cardiomyogenic differentiation marker Gata4. Scale bar, 20 μm. Data shown are representative of 3 independent experiments.

To investigate the effects of hHVS on cardiomyogenic differentiation of murine BM c-kit+ cells, we analyzed the expressions of cardiomyogenic differentiation-related markers, such as Nkx2.5, Gata4, and α-sarcomeric actin (α-SA) in cells growing on the hHVS and cells cultured without hHVS. On day 10 of the cell cultures, both reverse-transcription PCR (Fig. 4a) and semi-qPCR (Fig. 4b–d) revealed that the Nkx2.5, Gata4, and α-SA mRNA expressions were almost exclusively upregulated in cells growing on the hHVS. On day 20, semi-qPCR analysis showed that mRNA levels of the Nkx2.5, Gata4, and α-SA were significantly upregulated by 5.25-fold, 4.83-fold, and 25.67-fold, respectively, in BM c-kit+ cells growing on the hHVS, when compared with cells cultured without the hHVS (Fig. 4b–d). Immunohistochemical staining further showed that the expression of Gata4 by a fraction of BM c-kit+ cells growing on the hHVS (day 20) but not in the cells cultured without hHVS (Fig. 4e). These results demonstrate the capacity of the hHVS to promote cardiomyogenic differentiation of BM stem cells.

### hHVS and c-kit+ Cell-Seeded hHVS Improve Cardiac Repair *in Vivo*

**Figure 5:**
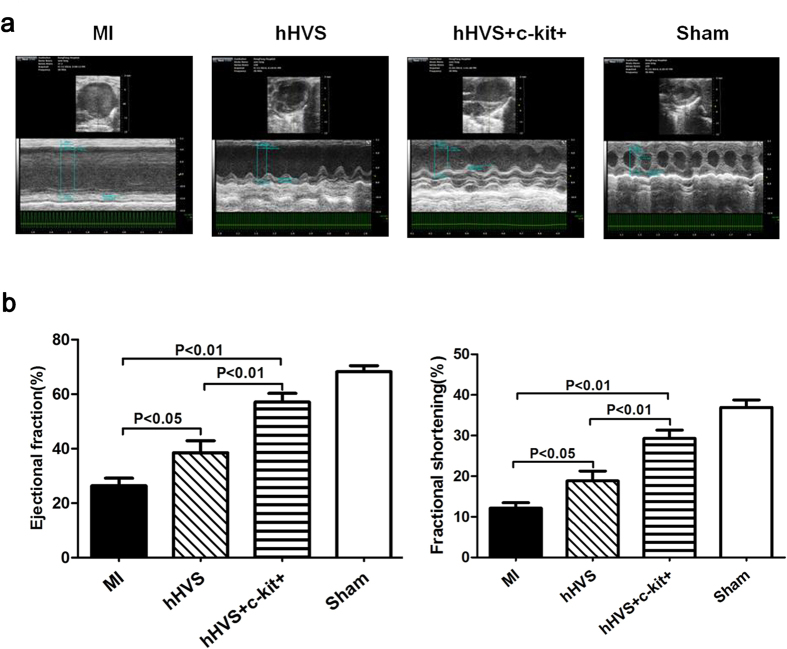
Echocardiographic examination 4 weeks after MI/implantation. (**a**) Representative echocardiography of MI (without patch implantation), hHVS (MI with implantation of hHVS alone), hHVS + c-kit+ (MI with implantation of c-kit+ cell-seeded hHVS), and sham groups. (**b**) Comparisons of ejection fraction (EF), fractional shortening (FS). MI, n = 10; hHVS, n = 10; hHVS + c-kit+, n = 10; sham, n = 7.

**Figure 6:**
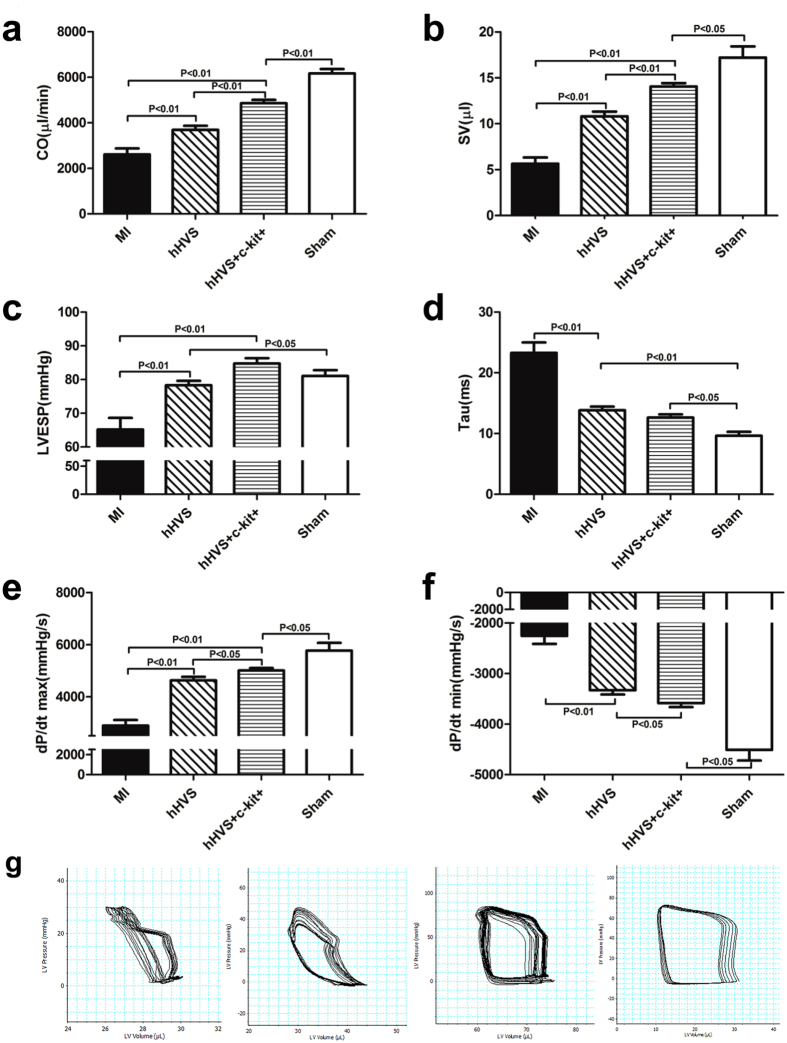
Hemodynamic measurements via a Millar pressure-volume catheter. (**a**–**f**) Calculated haemodynamic parameters shown as indices of pump function (cardiac output, CO; stroke volume, SV), systolic function (LV end-systolic pressure, LVESP; maximal peak rate of LV pressure, dP/dt max) and diastolic function (minimal peak rate of LV pressure, dP/dt min; relaxation time constant, Tau) in MI (without patch implantation), hHVS (MI with implantation of hHVS alone), hHVS + c-kit+ (MI with implantation of c-kit+ cell-seeded hHVS), and sham groups. (**g**) Pressure and volume signals were combined to construct the representative pressure–volume loops showing the pressure volume relationship in each group. Each loop represents one cardiac cycle. MI, n = 10; hHVS, n = 10; hHVS + c-kit+, n = 10; sham, n = 7.

Our results described earlier have clearly shown the enhanced regenerative potential of BM stem cells attached to the hHVS. This promoted us to investigate the *in vivo* effect of the hHVS for cardiac repair. To this end, we applied either the hHVS alone or BM c-kit+ cell-seeded hHVS to patch infarcted heart in a murine model of MI. The c-kit+ cell-seeded hHVS was obtained by growing post-infarct mouse BM c-kit+ cells (3 × 10^5^/cm^2^) onto hHVS for 10 days. Of note, transthoracic Doppler echocardiography showed that implantation of either hHVS alone or c-kit+ cell-seeded hHVS onto infarcted myocardium significantly increased ejection fraction (EF; from 25.3% to 38.3% or to 59%) and fractional shortening (FS; from 12.3% to 18.7% or to 30.4%), respectively, on day 28 post MI/implantation (Fig. 5; Supplementary Table 1). Importantly, the c-kit+ cell-seeded hHVS was even more efficient in improving cardiac function than the hHVS alone, as evidenced by additional significant reductions in systolic and diastolic indices, such as EF, FS, end-systolic LV inner dimension (LVIDs), end-diastolic LV inner dimension (LVIDd), LV end-systolic volume (LVESV) and LV end-diastolic volume (LVEDV) (Fig. 5; Supplementary Fig. S2, Supplementary Table 1). To further evaluate the cardiac effects of these cardiac patches, hemodynamic measurements via a Millar catheter were also monitored on day 28 post MI/implantation. Again, either the hHVS alone or c-kit+ cell-seeded hHVS drastically improved cardiac systolic and diastolic indices, as shown by the significantly increased cardiac output (CO), LV end-systolic pressure (LVESP) and maximal peak rate of LV pressure (dP/dt max), and by improved LV end-diastolic pressure (LVEDP), relaxation time constant (Tau) and minimal peak rate of LV pressure (dP/dt min) (Fig. 6; Supplementary Table 1). In addition, the c-kit+ cell-seeded hHVS was superior to hHVS alone in increasing CO, SV and LVESP, as well as in improving dP/dt max and dP/dt min (Fig. 6; Supplementary Table 1).

**Figure 7:**
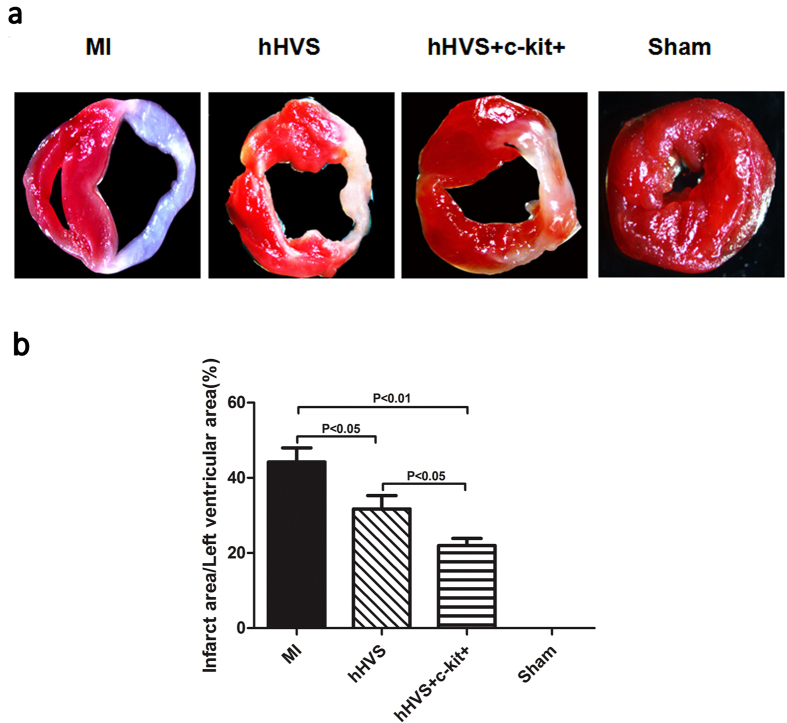
Measurement of infarct size. (**a**) Representative triphenyltetrazolium chloride (TTC) staining of heart sections showing the pale infarct region of MI (without patch implantation), hHVS (MI with implantation of hHVS alone), and hHVS + c-kit+ (MI with implantation of c-kit+ cell-seeded hHVS) groups. (**b**) Comparisons and statistical analysis of infarcted area among different groups. MI, n = 10; hHVS, n = 10; hHVS + c-kit+, n = 10; sham, n = 7.

To explore whether implantation of these cardiac patches could reduce cardiac injury, we evaluated the infarct size. The infarct size in MI mice was much smaller after implantation of the hHVS alone or c-kit+ cell-seeded hHVS than that without patch implantation (31.7% or 22.0% vs. 44.2%, respectively) (Fig. 7a). In line with the improvement of cardiac functional parameters, the infarct size in MI mice treated with c-kit+ cell-seeded hHVS was also significantly reduced, when compared with that treated with the hHVS alone (Fig. 7b). Moreover, the engrafted donor GFP+ cells were still detectable in periinfarct myocardium of MI mice 4 weeks after implantation of GFP+ c-kit+ cell-seeded hHVS (Supplementary Fig. S4), whereas an increased infiltration of CD45+ leukocytes into infarcted myocardium was not observed after implantation of the hHVS alone or c-kit+ cell-seeded hHVS (Supplementary Fig. S3).

Taken together, these results demonstrate that both the hHVS alone and c-kit+ cell-seeded hHVS improve cardiac performance and reduce cardiac injury while c-kit+ cell-seeded hHVS is superior to hHVS alone in mediating cardiac repair.

## Discussion

In this study, we have developed a human heart valve-derived scaffold (hHVS) for cardiac repair. Upon anchoring onto the hHVS, post-infarct murine BM c-kit+ cells exhibited an increased capacity for cell proliferation and cardiomyogenic differentiation *in vitro*. When used to patch infarcted heart in a murine model of MI, either the hHVS alone or c-kit+ cell-seeded hHVS significantly improved cardiac performance and reduced infarct size while c-kit+ cell-seeded hHVS was superior to the hHVS alone. These *in vitro* and *in vivo* observations provide the first evidence for translating the hHVS-based cardiac tissue engineering into clinical strategies to treat MI.

Cardiac tissue engineering using biomaterials with or without stem cell combination represents a promising therapeutic strategy for repairing infarcted heart and is now under intense investigation in translational cardiovascular research^11^,^20^,^21^. Theoretically, the ideal biomaterials for cardiac tissue engineering should provide the proper microenvironment of native myocardial extracellular matrix, allowing for functional cell-matrix interactions^20^,^22^. However, current cardiac tissue engineering is designed mainly based on natural and synthetic biomaterials^12^,^13^,^14^,^15^,^16^,^17^,^18^,^19^, which do not have the similar biochemical constituents and structural properties of natural myocardial extracellular matrix. In this study, we went a step further and for the first time successfully developed the hHVS, which improved cardiac performance and reduced infarct size when patching onto infarcted heart in MI mice, providing a clinically relevant novel scaffold for treating MI.

Transplantation of BM-derived stem cells into infarcted heart holds promise as a novel option for the treatment of MI; yet its therapeutic efficiency remains low^23^. Accumulating experimental studies indicate that the poor engraftment rate of transplanted cells into infarcted myocardium is the main cause^20^,^23^. Therefore, attempts from various researchers were made to enhance the engraftment of transplanted cells into infarcted myocardium^4^,^24^. In this study, we tested the engraftment of a well characterized BM stem cell population, c-kit+ cells, into the hHVS and analyzed whether hHVS engrafted with these c-kit+ cells (c-kit+ cell-seeded hHVS) has additional advantage when patching onto infarcted heart. To our knowledge, this is the first report about a direct engraftment of BM stem cells into decellularized native human myocardial scaffold. Of note, when used to patch infarcted heart in a murine model of MI, c-kit+ cell-seeded hHVS was superior to the hHVS alone in improving cardiac performance as well as in limiting infarct size. As both the hHVS alone and c-kit+ cell-seeded hHVS exerted beneficial effects in repairing infarcted heart the additional benefits of c-kit+ cell seeded hHVS over the hHVS alone could not be explained solely on the basis of the hHVS-mediated mechanisms, such as mechanical support of LV wall, provision of cardiac-specific signals, release of mitogenic/chemotactic signals for endogenous stem cells^20^. It seems that the engrafted c-kit+ cell-mediated regenerative/protective actions^1^,^2^,^3^,^5^ may contribute to those additional benefits of c-kit+ cell-seeded hHVS. Since the hHVS alone was able to promote proliferation of anchoring post-infarct BM c-kit+ stem cells, the enhanced regenerative/protective capacities of both exogenous and endogenous stem cells through cell-matrix interactions may account for the mechanisms of action by c-kit+ cell-seeded hHVS as well. The mechanisms by which the hHVS mediates stem cell proliferation are not clear. Emerging evidence has suggested that the extracellular matrix appears to influence the stem cell proliferation likely through physical interactions with cells, involving the mechanical properties and nanotopographic cues of the extracellular matrix, and the transmission of mechanical/biophysical factors to the cells^25^,^26^. An in-depth understanding of how physical signals influence the stem cell activity may help improve future strategies in constructing engineered cardiac patch. In the *in vivo* study, we failed to show the co-localization of cardiomyocyte markers or non cardiomyocyte markers in the engrafted GFP+ bone marrow cells although we have demonstrated an increased potential of these cells for cardiac differentiation *in vitro*. It may be argued that the engrafted bone marrow cells may act *in vivo* most likely via the cell fusion and/or paracrine mechanisms in the complex myocardial microenvironment^6^,^27^. In accordance with our current data, increasing evidence has indeed refuted the cardiac transdifferentiation of the engrafted bone marrow cells^28^. Various previous studies also suggest that the cell fusion and paracrine effects are the major mechanisms contributing to bone marrow cell-mediated cardiac repair^6^,^27^. The exact mechanisms that underlie the additional beneficial effects of c-kit+ cell-seeded hHVS merit further investigations.

The feasibility of translating native tissue-derived scaffolds into clinical strategies to treat MI remains challenging. Because each tissue has its own distinct mixture of extracellular matrix components that provides the unique cellular cues^20^,^29^, non-myocardial tissue-derived scaffolds such as urinary bladder matrix and small intestine submucosa (which form undesirable cartilage in the myocardium)^18^,^19^ may not be the suitable tissue sourcing for cardiac tissue engineering. With regard to the difficulties in finding the appropriate human myocardial tissues we here took the advantage of the availability of heart valve tissues (discarded during surgery) to serve as the realistic myocardial tissue sources. With the complete removal of cells from sliced human heart valve tissue, human immunogenic antigens in sliced acellular tissue should also be removed. Indeed, current data did not show any significant immune response and additional immune-mediated myocardial injury^30^ induced by implantation of xenogeneic hHVS in a murine model of MI. This indicates that the decellularized hHVS can be used as a biocompatible biomaterial for translational cardiac tissue engineering. As the availability of allogeneic valve scaffolds is also limited, future work may focus on xenogeneic valve scaffolds. A scaffold construct with strong tensile strength might be produced by stacking valve scaffolds^31^ or by the use of the electrospinning technique in the fabrication of hHVS-mimicking scaffolds^32^.

Limitations of this study should also be mentioned. The assessment of myogenic differentiation potential in bone marrow c-kit+ cells was limited to the overall cell content on the hHVS. Calculating the exact percentage of cells, which became cardiomyocytes, and distinguishing other cell types were hampered by an unknown rate of cell proliferation and loss in the cellular composition after 15 days in culture.

In summary, we have successfully developed a human heart valve-derived scaffold (hHVS). We show that the hHVS was able to promote proliferation and cardiomyogenic differentiation of BM c-kit+ cells *in vitro*. When used to patch infarcted heart in a murine model of MI, both the hHVS and c-kit+ cell-seeded hHVS significantly improved cardiac performance, with the latter exerting a more profound effect, thus providing a clinically relevant novel scaffold for cardiac tissue engineering.

## Methods

### Acquirement and Preparation of hHVS

Human mitral valves were obtained during mitral valve replacement from patients with chordae tendinae rupture at Ren Ji Hospital. The hHVS was prepared by freeze-sectioning mitral valve tissues at thicknesses of 50 μm or 100 μm, then decellularized in 0.5% or 1% sodium dodecyl sulfate (SDS) (depending on the thickness of the scaffold) for 12 hours. Decellularized hHVS was extensively washed to remove residual SDS. After lyophilization in a vacuum freeze-drier (VirtisBenchtop 6.6, SP Industries, Gardiner, NY), the hHVS was disinfected in 75% ethanol for 2 hours. The study was approved by the ethics committee of Ren Ji Hospital (no. 2012027) and informed consent was obtained in accordance with the Declaration of Helsinki.

### Induction of MI Model in Mice

MI was induced in male C57BL/6 mice (Shanghai Slaccas laboratory Animal Center, China) and CAG-EGFP transgenic mice (Shanghai Biomodel Organism, China) at age of 6–8 weeks (20–25 g) as described^33^. Briefly, after left lateral thoracotomy, a suture was tightened around the proximal left anterior descending coronary artery. Sham-operated mice received the same procedure with exception of coronary ligature. Animal care and applications of experimental procedures complied with the Guide for the Care and Use of Laboratory Animals approved by the Local Research Ethics Committee at Ren Ji Hospital.

### Isolation and Culture of Murine BM c-kit+ Cells

BM was flushed from femurs and tibias into Hanks’ balance salt solution (Biowest, L0606) on day 3 after MI. BM mononuclear cells were isolated by density gradient sedimentation using Mouse 1× Lymphocyte Separation Medium (Dakewe Biotech, DKW33-R0100). The c-kit+ cells were positively selected using magnetic activated cell sorting system (Miltenyi, 130091224), and then cultured on the hHVS inserted in a 24-well plate or without hHVS in Iscove’s Modified Dulbecco’s Medium (12440–053, Gibco) supplemented with 10% (v/v) fetal bovine serum (Biowest, S1810) for 1–20 days. The purity of sorted c-kit+ cells was analyzed by an Accuri C6 flow cytometer with CFlow Plus Software (BD, USA).

### Scanning Electron Microscope

The hHVS was rinsed with phosphate-buffered saline and fixed overnight in 0.05% glutaraldehyde at 4 °C. After dehydration through graded series of ethanol, samples were critical-point dried and examined on a scanning electron microscope (SEM; JEOL-6380LV, Japan). For some experiments, the c-kit+ cell-seeded hHVS was first obtained by growing post-infarct murine BM c-kit+ cells (3 × 10^5^ cells/cm^2^) onto the hHVS for 10 days in culture and then followed the procedures as described earlier.

### Cell Proliferation Assay

Murine BM c-kit+ cells (3 × 10^5^ cells/well) were seeded on the hHVS inserted in a 24-well plate or cultured without hHVS. CellTiter 96® AQueous One Solution Regent (Promega, USA) was added to the cells and incubated at 37 °C for 4 hours on days 1, 3, 5, 7, 10, and 15. The absorbance (OD490 nm) was measured using Biotek Synergy™ HT Multi-Mode Microplate Reader (Biotek, USA).

### Histological and Immunofluorescent Staining

A fraction of cultured cells were fixed on days 10, 15, and 20 with 4% polyoxymethylene for 15 min at room temperature. The fixed cells were then permeabilized by using 0.25% triton-X for 20 min, blocked with 5% normal donkey serum (Jackson, USA) for 1 hour, and stained with mouse anti-Ki67 (BD, USA) or rabbit anti-Phospho-Histone H3 (Cell signal, USA) or goat anti-Gata4 (Santa Cruz, USA) at 4 °C overnight. Subsequently, cells were incubated with secondary antibody donkey anti-goat Alexa Fluor 488 (1:500; Invitrogen, USA), or anti-rabbit Alexa Fluor 488 (1:500; Invitrogen, USA) or anti-mouse Alexa 488 (1:500; Invitrogen, USA), at 30 °C for 30 min. Cell nucleus was stained with 4,6-diamidino-2-phenylindole (DAPI). For visual inspection, Nikon Eclipse Ti-S Inverted Fluorescent Microscope was used. For the staining of hHVS (before and after decellularization) and myocardial tissues, they were first fixed with 4% polyoxymethylene, embedded in Tissue-Tek OCT (Sakura Finetek, Japan) and cut into 5 μm sections, and then stained also with chicken anti-GFP or rat anti-CD45 (1:200; Abcam, USA), followed by incubation with secondary antibody Donkey Anti-Chicken FITC (1:500; Abcam, USA) or anti-rat Alexa 488 (1:500; Invitrogen, USA) or/and hematoxylin and eosin (H&E).

### RNA Extraction, Reverse Transcription-PCR

Total RNA was extracted using Quick-RNA^TM^ Micro Prep (Zymo, USA) according to manufacturer’s protocol. Reverse transcription was performed with 200 ng RNA in 10 μl of reaction reagent of GoScript™ Reverse Transcription System (Promega, USA). PCR was performed with 28 cycles of denaturing (94 °C, 30 s), annealing (55–60 °C, 30 s), and extension (72 °C, 45 s) with a final extension at 72 °C for 7 min. PCR products were visualized by electrophoresis on a 2% (w/v) agarose gel containing 0.1‰ GelGreen (Genview, USA) and analyzed using an Image lab system (Bio-Rad, USA). Primer sequences are as follows: *β-actin*: forward primer 5′-GGCTGTATTCCCCTCCATCG-3′ and reverse primer 5′-CCAGTTGGTAACAATGCCATGT-3′, product size: 154 bp; *Ki67*: forward primer 5′-CCTGCCCGACCCTACAAAAT-3′ and reverse primer 5′-TCCGCCGTCTTAAGGTAGGA-3′, product size: 313 bp; *Nkx2.5*: forward primer 5′-CAATGCCTATGGCTACAACGC-3′ and reverse primer 5′-GAGTCATCGCCCTTCTCCTAAA-3′, product size: 297 bp; *Gata4*: forward primer 5′-CCCTACCCAGCCTACATGG-3′ and reverse primer 5′-ACATATCGAGATTGGGGTGTCT-3′, product size 139 bp; alpha-sarcomeric actin (α-SA): forward primer 5′-CAGCCCTCTTTCATTGGT-3′ and reverse primer 5′-CTGCCTCATCATACTCTTGC-3′, product size: 310 bp.

### Epicardial Implantation of hHVS to Patch Infarcted Heart

The hHVS or c-kit+ cell-seeded hHVS was prepared by incubating the hHVS in the medium alone or by growing post-infarct murine BM c-kit+ cells (3 × 10^5^ cells/cm^2^) onto the hHVS for 10 days, respectively, before implantation. The hHVS or c-kit+ cell-seeded hHVS was sutured with 7–0 silk onto the epicardial surface over the infarcted zone and adjacent periinfarct myocardium immediately after the induction of MI.

### Measurement of Infarct Size

Infarct size was evaluated as described^30^. In brief, recipient mice were sacrificed four weeks after MI/implantation. The hearts were harvested and washed with saline, and then sliced into three transverse 2-mm sections from apex to base. Sections were incubated in 2% triphenyltetrazolium chloride (TTC; Sigma-Aldrich) prepared with 200 mM Tris buffer (pH 7.8) for 20 min at 37 °C in the dark and fixed with 4% paraformaldehyde. TTC stains viable myocardium a deep-red color, while necrotic myocardium is TTC negative and appears pale. Sections were photographed with a digital camera, and the pale infarct and the left ventricular areas on each section were calculated using IPP (Image-Pro-Plus7.0, IPP) imaging software. The infarct size was determined as the mean ratio of the infarct area and the left ventricular area from two contiguous sections which represent the major infarct region and was give in percentage.

### Evaluation of Cardiac Function

Four weeks after MI/Implantation, recipient mice were subjected to transthoracic echocardiography and pressure-volume (P/V) loop measurements (Millar Pressure-Volume System) to evaluate cardiac performance as described^24^,^34^. In brief, hearts were imaged 2-dimensionally in long-axis views at the level of the greatest LV diameter. The systolic and diastolic LV areas were measured at the same time. This view was used to position the M-mode cursor perpendicular to the LV anterior and posterior wall. *In vivo* hemodynamic analysis was performed using a 1.4 F pressure-volume catheter (SPR-839) 4 weeks after MI/Implantation. A panel of hemodynamic parameters were measured to determine cardiac systolic and diastolic function.

### Statistical Analysis

Results were depicted as mean ± SEM. Comparisons were analyzed by two-tailed Student’s *t* test or one-way ANOVA followed by Dunnett’s posthoc test. Differences were considered significant at *p* < 0.05.

## Additional Information

**How to cite this article**: Wan, L. *et al*. Human heart valve-derived scaffold improves cardiac repair in a murine model of myocardial infarction. *Sci. Rep.* **7**, 39988; doi: 10.1038/srep39988 (2017).

**Publisher's note:** Springer Nature remains neutral with regard to jurisdictional claims in published maps and institutional affiliations.

## Supplementary Material

Supplementary Information
