# Supplementary material for: Human heart valve-derived scaffold improves cardiac repair in a murine model of myocardial infarction
Source: Sci Rep. 2017 Jan 4;7:39988. doi: 10.1038/srep39988 (PMC5209673; doi:10.1038/srep39988)
Supplement: Supplementary Information [file srep39988-s1.pdf]

Title: Human heart valve-derived scaffold improves cardiac repair in a murine model of myocardial infarction

Authors: Long Wan, Yao Chen, Zhenhua Wang, Weijun Wang, Sebastian Schmull, Jun Dong, Song Xue, Hans Imboden, Jun Li

Supplementary information

Figure S1

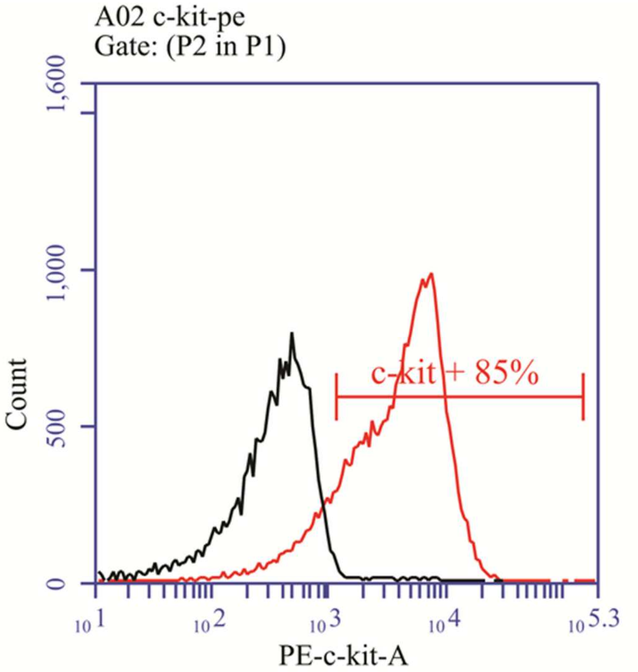

Supplementary Figure S1 | The purity of isolated murine BM c-kit+ cells. Murine BM was flushed from femurs and tibias on day 3 after MI. Following density gradient sedimentation, the c-kit+ cells were positively selected using magnetic activated cell sorting (MACS). The purity of isolated c-kit+ cells is around 85%, as determined by an Accuri C6 flow cytometer with CFlow Plus Software. Data shown are representative of 3 independent experiments.

Figure S2

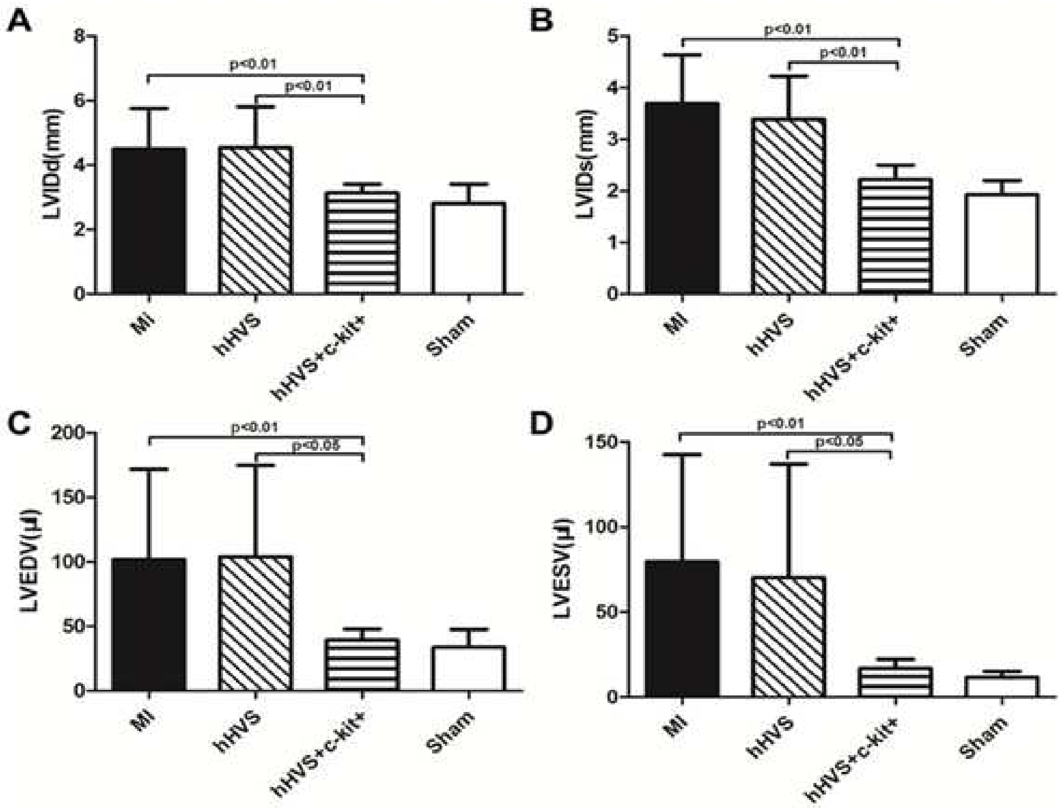

Supplementary Figure S2 | The c-kit+ cell-seeded hHVS is superior to hHVS alone in improving cardiac function. (A-D): Comparisons of echocardiographic parameters of MI (without patch implantation), hHVS (MI with implantation of the hHVS alone), hHVS + c-kit+ (MI with implantation of c-kit+ cell-seeded hHVS), and sham groups 4 weeks after MI/implantation. LVIDd, end-diastolic left ventricular dimension; LVIDs, end-systolic left ventricular dimension; LVEDV, left ventricular end-diastolic volume; LVESV, left ventricular end-systolic volume. MI, n=10; hHVS, n=10; hHVS + c-kit+, n=10; sham, n=7.

Figure S3

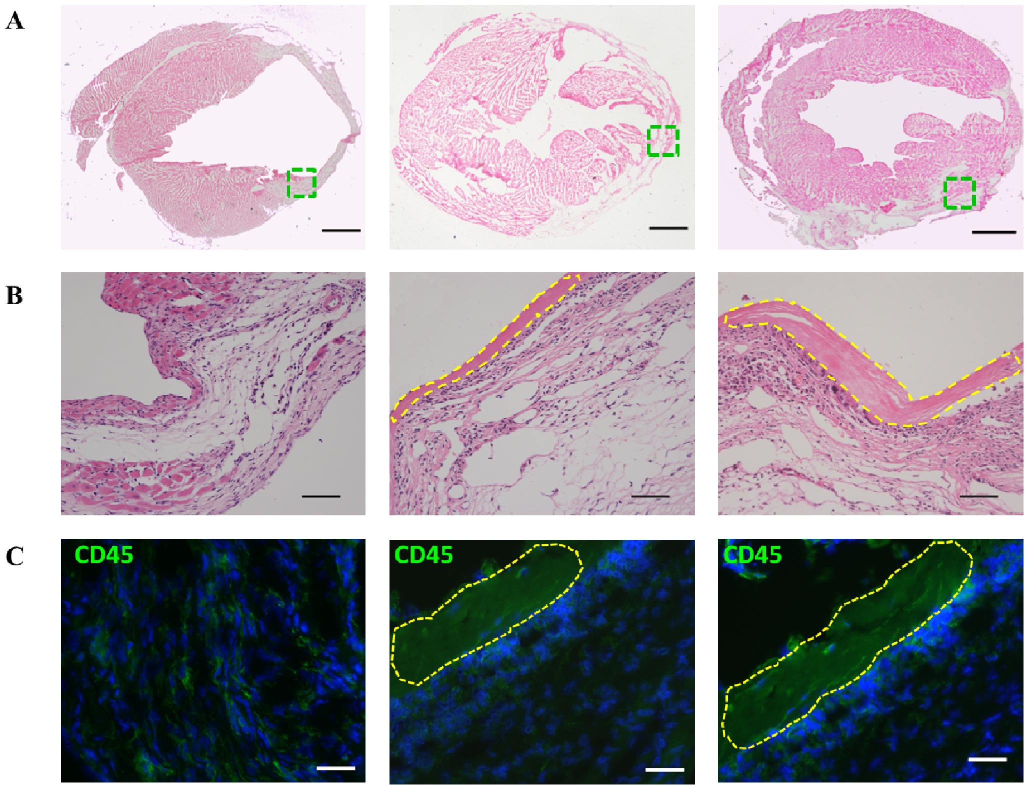

Supplementary Figure S3 | No increased infiltration of CD45+ leucocytes after implantation of hHVS or c-kit+ cell-seeded hHVS. HE staining (a, b) and immunofluncent staining (c) showing that an increased infiltration of CD45+ leucocytes (in green) into infarcted myocardium was not additionally induced 4 weeks after implantation of the hHVS alone or c-kit+ cell-seeded hHVS. Green and yellow dotted lines indicate the region analyzed for immunofluncent staining and implanted hHVS, respectively. (a), Scale bar, 1 mm; (b, c), Scale bar, 50 µm. Data shown are representative of 5 independent experiments.

Figure S4

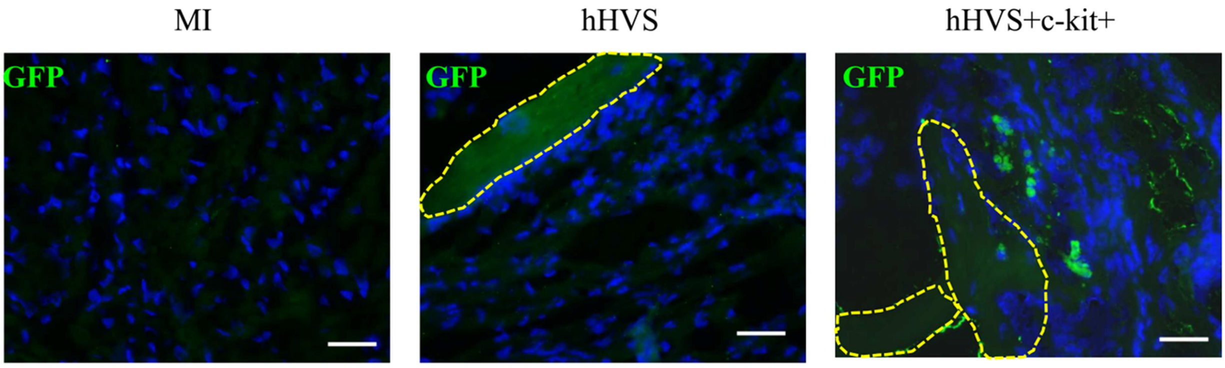

Supplementary Figure S4 | Detection of engrafted donor GFP+ cells in the periinfarct myocardia of recipient MI mice. Immunofluorescent staining of heart sections showing engrafted donor GFP+ cells (in green) surrounding the periinfarct myocardia of recipient MI mice 4 weeks after implantation of GFP+c-kit+ cell-seeded hHVS. The GFP+ cell is absent in the infarcted heart of MI mice with or without implantation of the hHVS. Yellow dotted lines indicate the implanted hHVS or GFP+c-kit+ cell-seeded hHVS. Data shown are representative of 5 independent experiments. Nuclei (in blue) are stained with DAPI; Scale bar, 50 μm.

Supplementary Table 1

Echocardiography and hemodynamic parameters 4 weeks after cardiac patch implantation

	MI	hHVS	hHVS+c-kit+	Sham
**Echocardiographic examination**				
EF (%)	25.27±2.64	38.27±2.57*	58.91±2.93**&&	68.20±2.27**&&$
FS (%)	12.27±1.46	18.71±2.54*	30.40±1.81**&&	37.15±1.76**&&$
LVIDd (mm)	5.12±0.40	3.91±0.40	3.14±0.09*&&	2.81±0.23*&&
LVIDs (mm)	4.49±0.46	3.23±0.23	2.22±0.09*&&	1.93±0.10**&&
LV mass (mg)	67.93±12.02	80.90±14.58	40.35±2.25*&	42.83±5.02*
LVEDV (μl)	102.00±22.04	103.90±22.38	39.47±2.67**&	33.83±5.17**&
LVESV (μl)	79.67±19.87	70.27±21.10	16.98±1.65**&	11.32±1.90**&$
**Hemodynamic parameters**				
CO (μl/min)	2609 ± 265.3	3689 ± 180.0**	4860 ± 148.0**&&	6168 ± 198.6**&&$$
SV (μl)	5.64 ± 0.68	10.80 ± 0.53**	14.06 ± 0.36**&&	17.20 ± 1.23**&&$
LVESP (mmHg)	65.15 ± 3.48	78.33 ± 1.30**	84.76 ± 1.57**&	81.08± 1.67**&&
LVEDP (mmHg)	7.08 ± 0.89	5.78 ± 0.79**	5.26 ± 0.85**	3.68 ± 1.52**&&$
dP/dt max (mmHg/s)	2892 ± 218.6	4632 ± 132.7**	5010 ± 89.16**&	5769 ± 297.7**&
dP/dt min (mmHg/s)	-2253 ± 159.0	-3328 ± 85.40**	-3585 ± 76.36**	-4509 ± 208.8**&$
Tau (ms)	23.28 ± 1.72	13.84 ± 0.59**	12.63 ± 0.54**	9.65 ± 0.65**&&$

Supplementary Table 1. Echocardiography and hemodynamic parameters (Millar pressure-volume catheter) of MI (without patch implantation), hHVS (MI with implantation of the hHVS alone), hHVS + c-kit+ (MI with implantation of c-kit+ cell-seeded hHVS), and sham groups 4 weeks after MI/implantation. CO= cardiac output; dP/dt max= maximal rate of rise of left ventricular diastolic pressure; dP/dt min= minimal rate of rise of left ventricular diastolic pressure; LVEDV= left ventricular end-diastolic volume; LVEDP=left ventricular end-diastolic pressure; LVESP= left ventricular end-systolic pressure; LV mass= left ventricular mass; LVESV= left ventricular end-systolic volume; LVIDd= end-diastolic left ventricular dimension, LVIDs= end-systolic left ventricular dimension; SV= stroke volume; Tau=left ventricular

relaxation time constant. MI, n=10; hHVS, n=10; hHVS + c-kit+, n=10; sham, n=7; **p*<0.05, ***p*<0.01 vs. MI; [&]*p*<0.05, [&&]*p*<0.01 vs. hHVS; [$]*p*<0.05, [$$]*p*<0.01 vs. hHVS + c-kit+.
